# The Shono-type electroorganic oxidation of unfunctionalised amides. Carbon–carbon bond formation via electrogenerated *N*-acyliminium ions

**DOI:** 10.3762/bjoc.10.323

**Published:** 2014-12-18

**Authors:** Alan M Jones, Craig E Banks

**Affiliations:** 1 Manchester Metropolitan University, Faculty of Science and Engineering, School of Science and the Environment, Division of Chemistry and Environmental Science, John Dalton Building, Chester Street, Manchester, M1 5GD, UK

**Keywords:** anodic oxidation, electrochemistry, electroorganic, electrosynthesis, *N*-acyliminium ions, natural products, non-Kolbe oxidation, peptidomimetics, Shono oxidation, synthesis

## Abstract

*N*-acyliminium ions are useful reactive synthetic intermediates in a variety of important carbon–carbon bond forming and cyclisation strategies in organic chemistry. The advent of an electrochemical anodic oxidation of unfunctionalised amides, more commonly known as the Shono oxidation, has provided a complementary route to the C–H activation of low reactivity intermediates. In this article, containing over 100 references, we highlight the development of the Shono-type oxidations from the original direct electrolysis methods, to the use of electroauxiliaries before arriving at indirect electrolysis methodologies. We also highlight new technologies and techniques applied to this area of electrosynthesis. We conclude with the use of this electrosynthetic approach to challenging syntheses of natural products and other complex structures for biological evaluation discussing recent technological developments in electroorganic techniques and future directions.

## Review

### *N*-Acyliminium ions are synthetically versatile

*N*-Acyliminium ions [1–2] have a long and distinguished history in organic chemistry being important components in carbon–carbon bond forming reactions and in the generation of cyclic and heterocyclic ring systems through such classic named reactions as the Pictet–Spengler and Diels–Alder [3–4]. There have been many excellent and comprehensive reviews on the art of electroorganic synthesis and the reader is directed to these articles for a thorough background and insight into the many facets of electrosynthesis [5–10]. In this review, we focus upon the development and application of the Shono-type electrochemical oxidation of unfunctionalised amides (last comprehensively reviewed in 1984 by Prof. T. Shono) [10] to *N*-acyliminium ion intermediates and their application to synthetic challenges.

### The Shono electrooxidation route to *N*-acyliminium intermediates

**Scheme 1:**
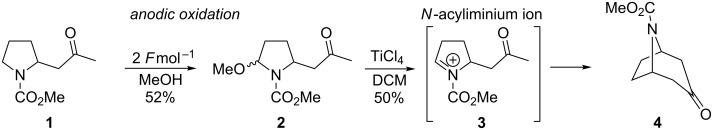
Application of anodic oxidation to the generation of new carbon-carbon bonds [11].

Shono and colleagues reported the first direct electrochemical anodic oxidation of an α-methylene group to an amide (or carbamate) to generate a new carbon–carbon bond via an anodic methoxylation step and Lewis acid mediated generation of an *N*-acyliminium ion reactive intermediate; Scheme 1 overviews such a process [11].

**Scheme 2:**
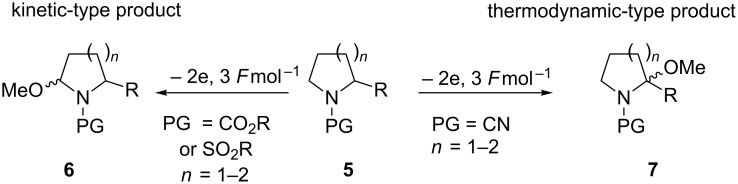
The influence of the amino protecting group on the “kinetic” and “thermodynamic” anodic methoxylation [16].

Although the anodic oxidation/alkoxylation of amides pre-dates this work [12–13], Shono showed the synthetic utility of combining an electroorganic step with key carbon–carbon bond forming reactions required in synthetic organic chemistry. The key anodic methoxylation is operationally straightforward with a standard electrochemical set-up using carbon electrodes and is well documented [14–15]. The anodic methoxylation of unsymmetrical amides raises a key question regarding the regioselectivity of the products formed. Onomura and colleagues have detailed the influence of the protecting group on nitrogen on a series of cyclic amines on the product formed. It was argued the protecting group would influence and stabilise the *N*-acyliminium ion formed, therefore altering the regioselectivity of the product obtained. It was found that in all cases (e.g., carbonyl or sulfonyl-based protecting groups and ring size, *n* = 1 or 2) the kinetic-type product was exclusively formed except when the protecting group (PG) was changed to a cyano group the thermodynamic-type product was the dominant product formed (see Scheme 2) [16]. Looking more closely at these examples (PG = CN), it was found that increasing the size of the R group had little effect in adjusting the kinetic/thermodynamic ratio when the ring size was *n* = 1. The kinetic/thermodynamic ratio became more pronounced when *n* = 2 and the thermodynamic product was formed exclusively when large R groups were used (for example, phenyl).

**Scheme 3:**
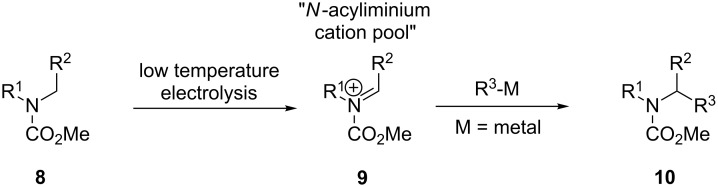
Example of the application of the cation pool method [17].

The conventional two-step electrochemical procedure for the generation of the *N*-acyliminium ion and trapping with solvent (e.g., methanol), regenerating the *N*-acyliminium ion through treatment with a Lewis acid (quenching with the nucleophile) can be overridden by the “cation pool” method [17]. The cation pool methodology relies on the low temperature electrolysis of carbamates to accumulate the *N*-acyliminium ion reactive intermediate then under non-oxidative conditions the nucleophile is introduced. Importantly, the nucleophile cannot be present when the *N*-acyliminium ion is being formed under electrochemical conditions, as in most cases it is easier to oxidise the nucleophile than the amide precursor. This circumvents the need to prepare, trap and release the *N*-acyliminium cation under more favourable conditions, allowing the direct α-alkylation or arylation of carbamates (Scheme 3); the cation pool method has been extensively studied [17–31].

### The use of electroauxiliaries

**Scheme 4:**
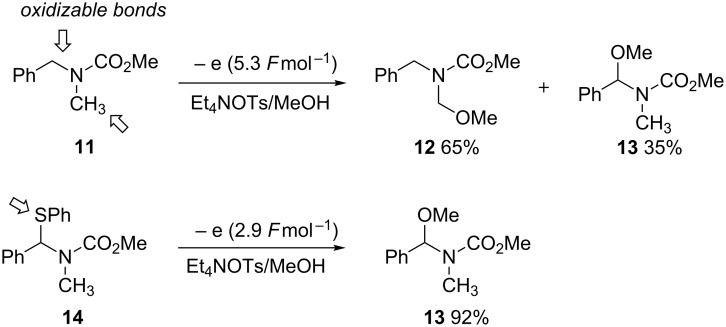
A thiophenyl electroauxiliary allows for regioselective anodic oxidation [32].

The concept of electroauxiliaries has proven useful for developing and expanding the scope of the Shono-type oxidation of carbamates; electroauxiliaries activate organic compounds towards electron transfer controlling the fate of the generated reactive intermediates and assist in the formation of the desired products. Tin, silicon or sulfur-based electroauxiliaries have proved useful in this endeavour. Yoshida and co-workers developed an organothio electroauxiliary that is selectively cleaved under anodic oxidation conditions [32–33]. Although the introduction of an electroauxiliary requires an additional synthetic step to prepare, the resulting carbon–tin, carbon–silicon or carbon–sulfur bond has a less positive oxidation potential than an unfunctionalised carbamate. Therefore, exquisite control can be exerted on the introduction of the nucleophile to the C–X bond that contains the electroauxiliary (Scheme 4).

**Scheme 5:**
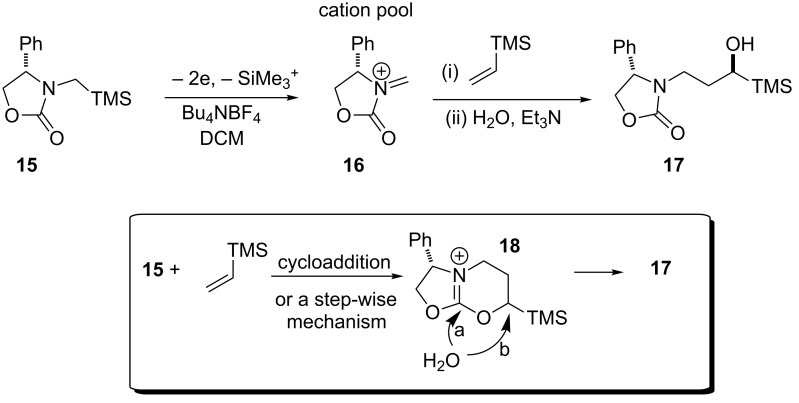
A diastereoselective cation carbohydroxylation reaction and postulated intermediate **18** [18].

Similarly, α-silyl-carbamates undergo low potential anodic oxidation reactions with complete regiocontrol and in special cases diastereoselectivity [34–35]. Interestingly, a porous graphite felt anode and a stainless steel cathode were utilised in a flow cell set-up [35]. An important example of the scope and synthetic potential of the silyl electroauxiliary approach was reported by Yoshida (Scheme 5) in combination with a chiral auxiliary a highly diastereoselective cationic carbohydroxylation occurred [18]. The proposed formation of a bicyclic intermediate **18** resulting from a cycloaddition reaction would enhance the diastereoselectivity of the hydration step compared to an acyclic intermediate that would be formed in the step-wise mechanism to **18**. The authors’ suggest the step-wise mechanism does play a role in reducing the diastereoselectivity of the reaction when the alkene is aryl- or alkyl-substituted. The hydration step can occur via pathway *a* or *b* to afford **17** (dr ca. 3:2).

**Scheme 6:**
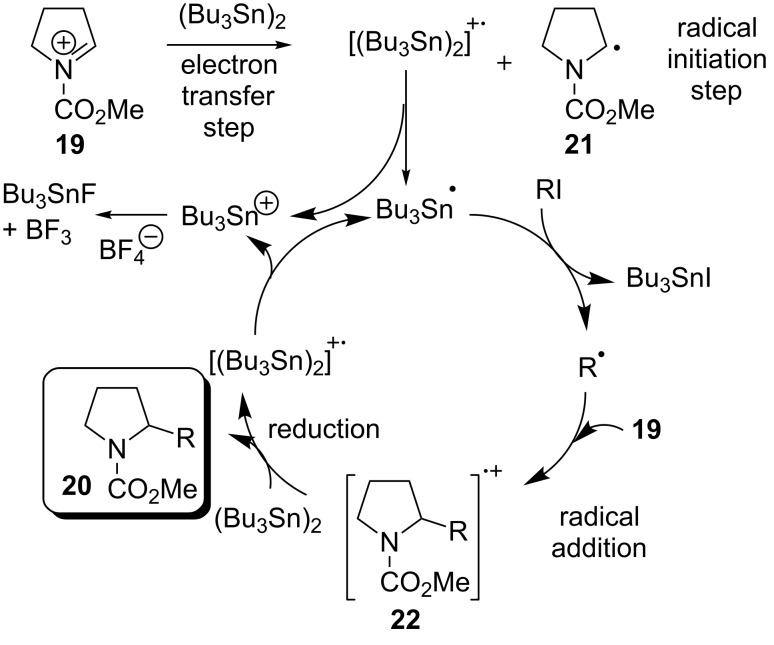
A radical addition and electron transfer reaction of *N*-acyliminium ions generated electrosynthetically [19].

The *N*-acyliminium ions formed during an anodic oxidation of a silyl auxiliary can be coupled to a radical pathway using a radical initiating agent such as distannane (Scheme 6). This allows the *N*-acyliminium ion to react with an alkyl halide to generate the typical carbon–carbon products of the Shono oxidation [19,27–28] Examples of reactions with activated olefins have been reported using the generation of carbon free radicals from the cation pool method [24–25].

### Indirect electrolysis methods

**Scheme 7:**
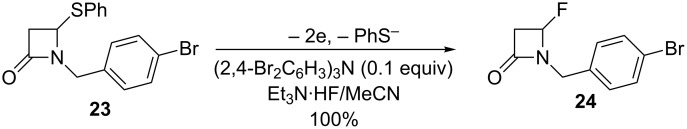
Catalytic indirect anodic fluorodesulfurization reaction [37].

The only indirect anodic oxidation method to perform the Shono-oxidation with a thiophenyl electroauxiliary has been reported by Fuchigami and co-workers [36]. Using a catalytic triarylamine redox mediator, anodic fluorodesulfurization occurred (Scheme 7). Direct Shono-type fluorination of an α-carbon to an amide has also been reported [37].

### Technical advances in the Shono electroxidation reaction

**Figure 1:**
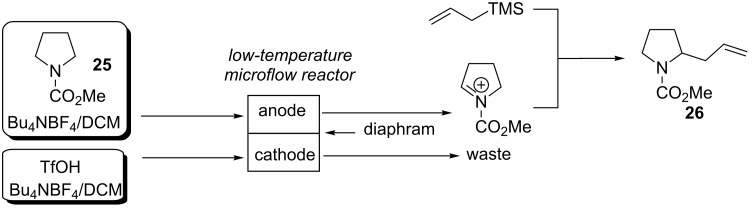
Schematic of a cation flow system and also shown is the electrochemical microflow reactor reported by Suga et al. Figure redrawn from reference [30].

The “cation-pool” method for the generation of *N*-acyliminium ions [17–31] has been adapted to efficiently generate novel small molecules in an electrochemical microflow system [26,30]. Combining the advantages of the cation-pool method with microflow technologies has enabled the concept of “cation flow” systems (Figure 1). In this arrangement a porous carbon felt is used as an anode and a platinum wire coil is used as the cathode; the flow cell is placed into a dry ice bath to maintain the −78 °C temperature required. Cation flow systems can be used to prepare individual molecules and combined with the concept of combinatorial chemistry to generate libraries of compounds.

**Figure 2:**
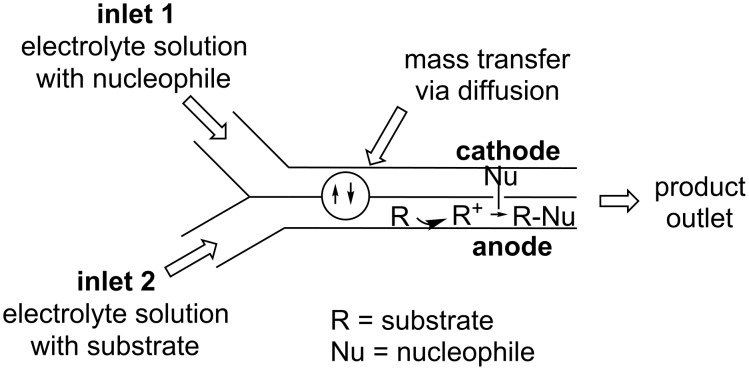
Example of a parallel laminar flow set-up. Figure redrawn from reference [38].

A further advance to the micro-flow reactor strategy is to use a parallel laminar flow set-up (Figure 2 overviews such an experimental design) [38–39]. Due to the small size of the flow channel when two liquids are injected the liquid–liquid contact area will remain stable and laminar, where only contact between anodically generated carbocations occurs by mass transfer diffusion across the liquid–liquid contact area. Therefore, one liquid can be oxidized and the other liquid containing the nucleophile can intercept the *N*-acyliminium ion formed in the microflow reactor (when the two sides of the channel are anode and cathode) (viz. Figure 2).

**Figure 3:**
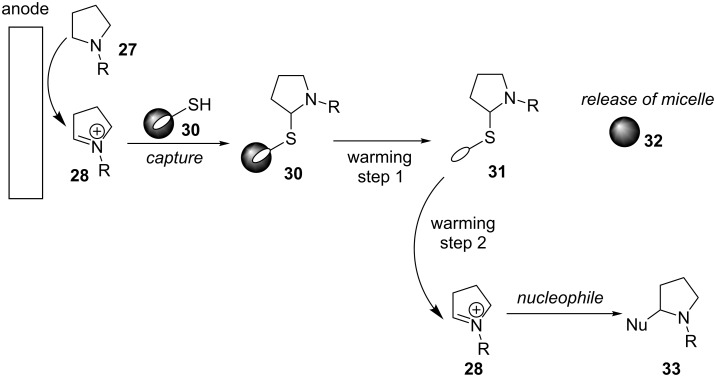
A catch and release cation pool method [42].

This technique of microflow mixing can also be applied to the synthesis of polymers [29]. A single channel miniaturised microfluidic electrolysis cell that is modular with other microfluidic techniques has now been developed to perform anodic methoxylation reactions [40]. Microflow mixing can confer other advantages such as increase electrode surface to reactant volume and reduced distance between electrodes. It has also been shown that electroorganic synthesis can be performed without supporting electrolyte in a microflow system [41]. If the cation pool method is unsuitable, for instance due to the instability of the cation formed, Chiba and co-workers have proposed a reversible capture method [42]. Using liquefiable micelle-like microparticles containing a thiomaleimide unit, *N*-acyliminium ions generated electroorganically can be intercepted and tagged in situ. The tagged and now stable “reactive intermediate” can then be removed from the electrolyte solution. Warming the tagged “reactive intermediate” converts the micelle (eicosane, a thermosensitive alkane) from solid to liquid and releases the thiomaleimide tagged *N*-acyliminium ion. Further warming breaks the C–S bond and allowed the regeneration of the *N*-acyliminium ion in a new solution containing the desired nucleophile interceptor to immediately react with the *N*-acyliminium ion as it is released from its thiomaleimide tag (Figure 3).

**Scheme 8:**
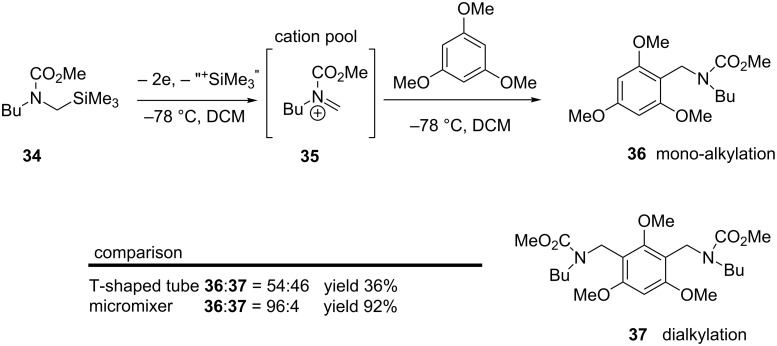
Micromixing effects on yield 92% vs 36% and ratio of alkylation products [43].

A micromixer has been used to generate an *N*-acyliminium ion pool and under Friedel–Crafts conditions mono-alkylation products are formed (Scheme 8) [43–44]. A problem with conventional Friedel–Crafts alkylation is the mono-alkylated product is more reactive than the starting material making di- and tri-alkylation products more likely. Improved yields and ratios of up to 96:4 mono-alkylated to dialkylated were observed using the cation pool micromixing strategy.

**Figure 4:**
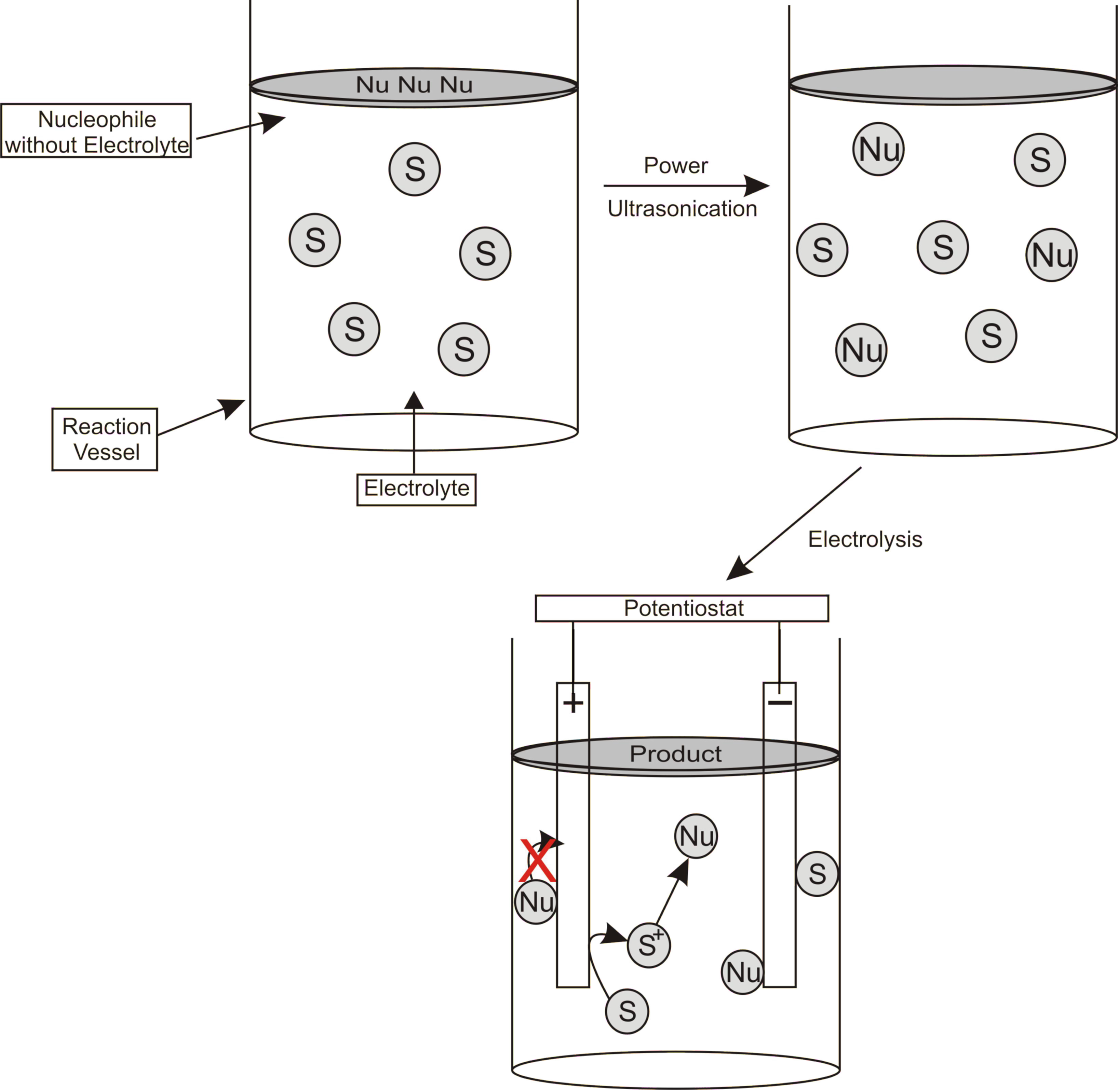
Schematic illustration of the anodic substitution reaction system using acoustic emulsification. Figure redrawn from reference [45].

Acoustic emulsification can be used when the desired nucleophile for an anodic methoxylation reaction which is insoluble in the electrolyte [45–46]. Figure 4 depicts the experimental set-up where the nucleophile is insoluble in an electrolytic medium and is dispersed as sub-micrometre sized droplets by the application of power ultrasound; such an approach results in a high interfacial liquid–liquid area of the sono-emulsion and trapping of the product.

Power ultrasound (20 kHz) induced a “temporarily soluble” droplet of the nucleophile that can then intercept the *N*-acyliminium ion. Electrosynthesis have been adapted to use solid-supported bases [47–51], performing the electrosynthesis on compounds directly attached to a catch and release solid-support [52], or having the nucleophile solid-supported [53]. The use of ionic liquids as a green electrolyte/solvent [54] and using solar power to provide the electrical current [55] are some of the recent additions to make electrosynthesis even more environmentally friendly. Further advances have been made using spatially addressable electrolysis platform’s (SAEP) [56]. This technique has been used to prepare both parallel and combinatorial libraries using Shono-type oxidation on a microarray. Some technical aspects of anodic alkoxylation have been patented [57].

### The use of the Shono-type electrooxidation in multiple branches of synthetic organic chemistry

**Scheme 9:**
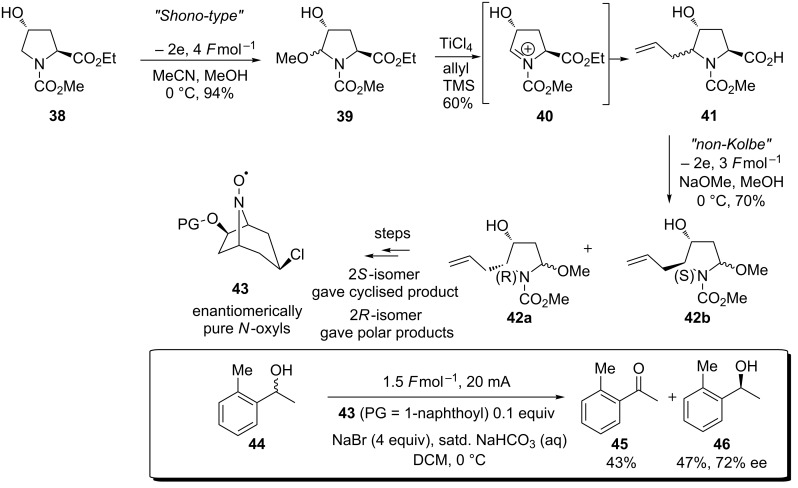
Electrooxidation to prepare a chiral oxidation mediator and application to the kinetic resolution of *sec*-alcohols [58].

The enantioselective electrooxidation of *sec*-alcohols mediated by azabicyclo-*N*-oxyls has been reported by Onomura and colleagues [58–59]. The azabicyclo-*N*-oxyl oxidation mediators were themselves prepared by anodic methoxylation. A chiral example of the azabicylco-*N*-oxyl was employed to kinetically resolve racemic *sec*-alcohols (Scheme 9).

The preparation of 1-(*N*-acylamino)alkyl sulfones from the anodic electrooxidation of non-Kolbe or Shono-type precursors affords the expected α-methoxyl products. Treatment with triphenylphosphonium salts and sodium aryl sulfinates afforded stable crystalline precursors of *N*-acyliminium ions that are activated by base [60]. The anodic methoxylation products of unfunctionalised amides can be converted to carbonyl compounds (aldehydes or esters) by treatment with cobaltacene [61] or be used as starting materials for the Morita–Baylis–Hillman-type reaction [62]. Anodic methoxylation can be combined with biocatalytic approaches to prepare stereodivergent 4-hydroxypiperidines [63]. 3-Hydroxy-6-substituted piperidines inaccessible by conventional synthesis approaches can also be effectively synthesised by anodic methoxylation [64].

**Scheme 10:**
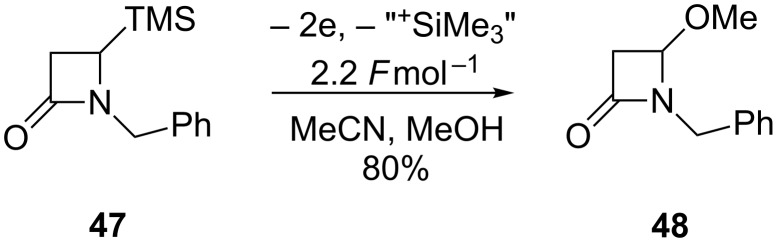
Electrooxidation reactions on 4-membered ring systems [68].

The scope of the anodic methoxylation has so far been limited to either acyclic or 4–6-membered ring sizes, the use of electrosynthetic approaches can also be applied to larger 7-membered ring systems, albeit less frequently [65–66]. β-Lactams (4-membered rings) undergo anodic oxidation of the carbon–silicon bond (when an electroauxiliary is present) or direct carbon–hydrogen bond fission to afford the α-methoxylated product (Scheme 10) [67–68].

**Figure 5:**
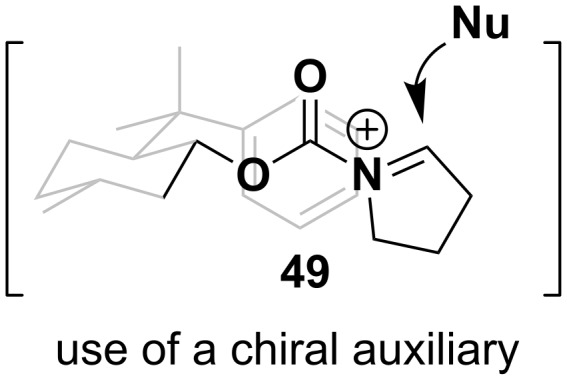
Example of a chiral auxiliary Shono-oxidation intermediate [69].

The direct anodic oxidation reaction to afford the *N*-acyliminium ion can be intercepted with a carbon nucleophile enantioselectively when a chiral auxiliary is attached either to the carbamate or amide (Figure 5) [69–71] (using a platinum anode and tungsten cathode electrochemical set-up) [69] or the use of Cu-PyBox chiral ligand systems [72].

**Scheme 11:**
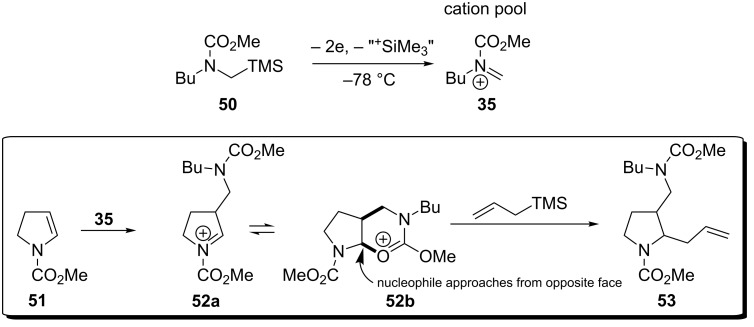
An electrochemical multicomponent reaction where a carbon felt anode and platinum cathode were utilised and carried out at −78 °C [22].

The cation pool method can be adapted to a multicomponent reaction (MCR) when an *N*-acyliminium ion is intercepted by an enamine [22]. Although the electrochemically generated *N*-acyliminium ion has reacted, the enamine generated a second *N*-acyliminium which in turn can be intercepted by another carbon nucleophile (Scheme 11). It was postulated that intermediate **52a** could cyclise to the bicyclic system **52b**. It proved difficult to determine the *cis/trans* relationship in many examples due to the presence of rotameric forms, however the products of phenyl magnesiumbromide were identified to be *trans* presumably due to opposite face attack of intermediate **52b**.

**Scheme 12:**
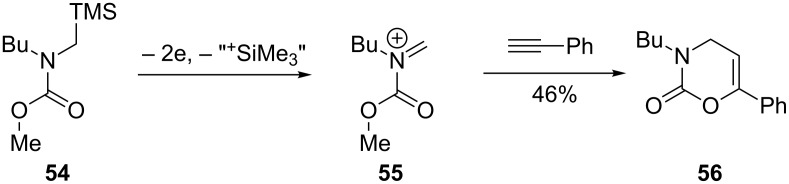
Preparation of dienes using the Shono oxidation [23].

Bicyclic lactams [73] and tricyclic systems [74] have also been prepared using the anodic oxidation route. Possibly, one of the most important uses of the Shono oxidation has been in the development of the [4 + 2] cycloaddition, more commonly known as the Diels–Alder reaction for the controlled preparation of *N*-acyliminium ions to react with dienophiles (Scheme 12) [21,23]. Another facet of this reaction was the controlled application of micro-mixing resulted in a significant improvement in isolated yield of the desired cycloadduct compared to batch synthesis (79% vs 20–57%).

The electrochemical version of the Diels–Alder reaction is gaining in popularity with an elegant synthesis of the natural product, kingianin A, recently published by the Moses group, albeit not through a Shono or non-Kolbe mechanism [75].

**Scheme 13:**
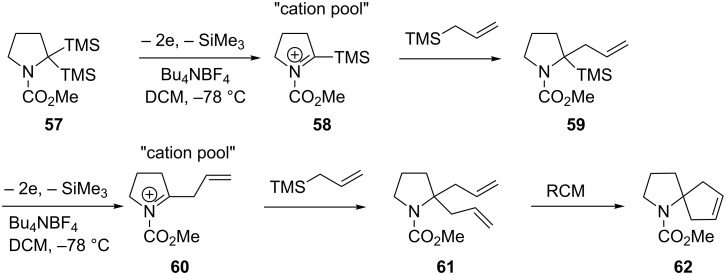
Combination of an electroauxiliary mediated anodic oxidation and RCM to afford *spiro*cyclic compounds [76].

The Shono-type electrooxidation has been used to prepare spirocyclic compounds using a double silyl electroauxiliary approach (Scheme 13) [76–77]. The ability to introduce two carbon–carbon bonds on to the same α-carbon to prepare spirocyclic systems is a challenge, yet the application of electrochemistry in tandem with ring closing metathesis (RCM) readily achieved this feat.

### The use of the Shono-type electrooxidation in natural product synthesis

**Scheme 14:**
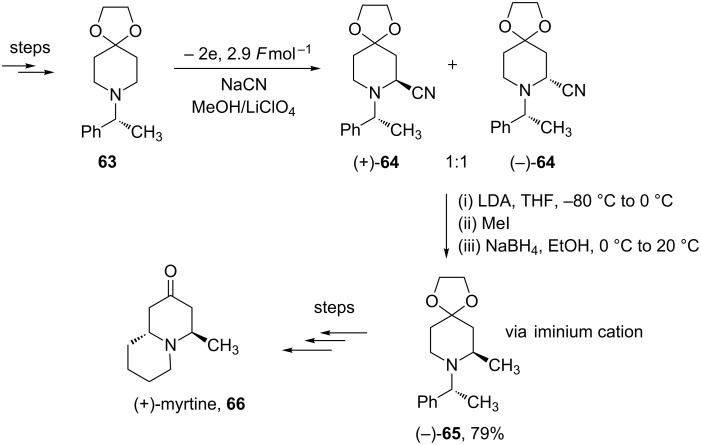
Total synthesis of (+)-myrtine (**66**) using an electrochemical approach [78].

The synthesis of natural products is considered a good test of the synthetic potential of a new reaction. The Shono-type oxidation has proved itself in the following syntheses. Hurvois and colleagues have reported an electrochemical asymmetric synthesis of (+)-myrtine (**66**) as shown in Scheme 14 [78]. (+)-Myrtine (**66**) is a member of the *trans*-4,9a-quinolizidin-2-one family, originally isolated from *Vaccinium myrtillis* (Ericaceae). The synthesis relied on the introduction of a cyano group followed by α-deprotonation by LDA and installation of a methyl group. Reductive decyanation of the α-amino nitrile, reinstalled the *N*-acyliminium ion. Reduction of the *N*-acyliminium afforded the major diastereoisomer as shown in Scheme 14 in 79% after column chromatography. Further synthetic modification of this key intermediate afforded the total synthesis of (+)-myrtine (**66**) in a further 5 steps in an overall yield of 30%.

**Scheme 15:**
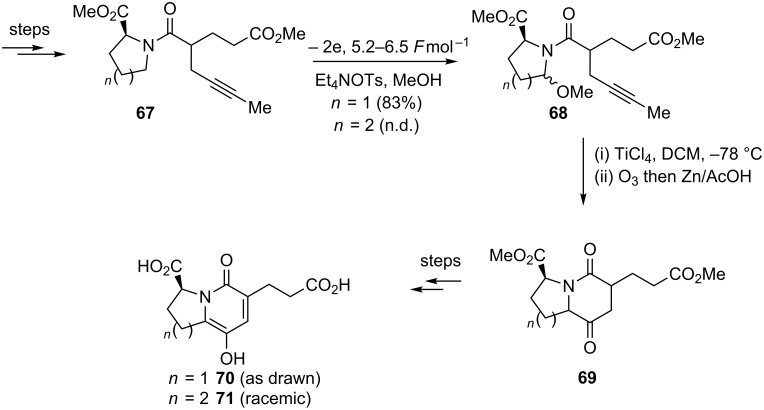
Total synthesis of (−)-A58365A (**70**) and (±)-A58365B (**71**) [79].

The Moeller research group has used an anodic amide oxidation for the total synthesis of the angiotensin-converting enzyme inhibitors, (−)-A58365A (**70**) and (±)-A58365B (**71**) (Scheme 15) [79]. This synthesis highlighted the power of the anodic amide oxidation-*N*-acyliminium ion cyclisation strategy in the presence of a disubstituted acetylene nucleophile. Anodic oxidation proceeded in high yield and a smooth cyclisation of the pendant acetylene nucleophile was triggered by treatment with titanium tetrachloride. Ozonolysis of the chloromethyl alkene intermediate afforded the carbonyl compound and the 5,6 and 6,6-ring systems of the target compounds.

**Scheme 16:**
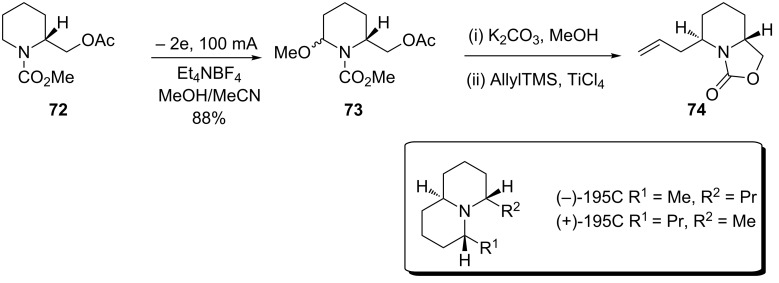
Anodic oxidation used in the preparation of the poison frog alkaloid 195C [80].

Toyooka and co-workers have designed a route to both enantiomers of the quinolizidine poison frog alkaloid 195C. Key to the success of their synthetic endeavour was the preparation via direct anodic oxidation of intermediate **73** (Scheme 16) [80]. 195C had never before been prepared enantioselectively and the first total synthesis of 195C utilised a key asymmetric Shono oxidation step.

**Scheme 17:**
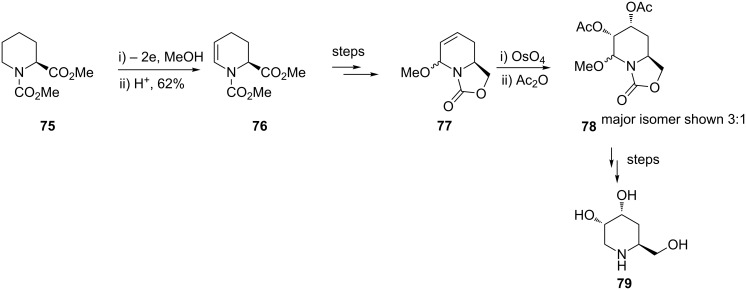
Preparation of iminosugars using an electrochemical approach [81].

Electrosynthesis using anodic oxidation has also been applied to the α-methylene of an amide for the preparation of iminosugars [81–82]. Iminosugars have shown a variety of biological effects including inhibiting glycosidases and glycoprotein-processing enzymes. Onomura and Matsumura and colleagues have used the anodic methoxylation and mild acid treatment strategy to prepare the initial starting materials in the synthetic campaign (Scheme 17).

**Scheme 18:**
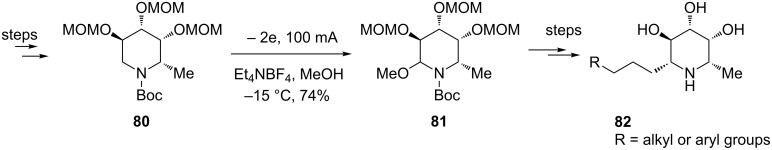
The electrosynthetic preparation of α-L-fucosidase inhibitors [84–85].

The Shono-type oxidation of unfunctionalised amides has been applied to the synthesis of inhibitors of a variety of biological targets [83], in particular α-L-fucosidase [84–85]. Toyooka and co-workers also applied anodic methoxylation to prepare iminosugars as potent inhibitors of α-L-fucosidase, an important target in the inflammation response (Scheme 18). The α-methoxy group introduced could then be intercepted via an *N*-acyliminium ion intermediate with a variety of C–C bond forming reagents. The compounds prepared were interrogated for bioactivity against α-L-fucosidase and related targets and IC_50_’s of as low as 1 nM were reported for α-L-fucosidase with limited off-target activity.

**Scheme 19:**
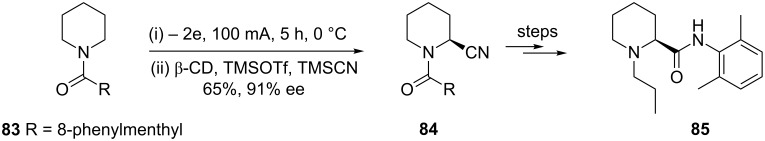
Enantioselective synthesis of the anaesthetic ropivacaine **85** [71].

The total synthesis of the anaesthetic ropivacaine (**85**) was accomplished enantioselectively using as its key step a direct anodic oxidation to prepare at low temperatures a cation pool of *N*-acyliminium ions that were intercepted with cyanide [71]. The enantioselectivity induced in this step was as a result of using a chiral auxiliary, 8-phenylmenthyl attached to the carbamate (Scheme 19).

Other natural product syntheses have used the anodic oxidation approach, often as the first step in a synthesis campaign to functionalise a pyrrolidine or piperidine carbamate [86–87].

**Scheme 20:**
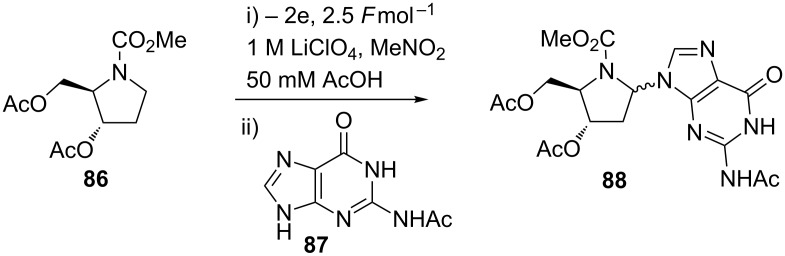
The preparation of synthetically challenging aza-nucleosides employing an electrochemical step [88].

A lithium perchlorate–nitromethane system was used to prepare electrochemically azanucleoside derivatives [88]. Unactivated prolinol derivatives underwent anodic oxidation to generate *N*-acyliminium ions that were intercepted by nucleophilic bases such as the nucleobases: protected cytosine, guanine **87**, adenine, and thymine to afford azanucleoside products such as **88** (Scheme 20).

### The use of the Shono-type electrooxidation in peptide and peptidomimetic chemistry

**Scheme 21:**
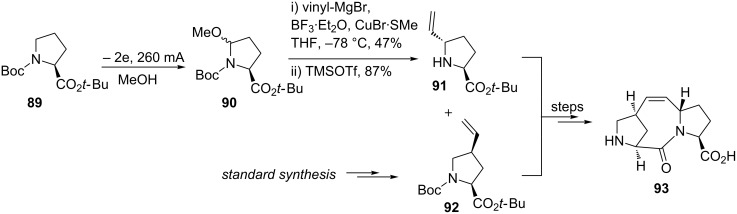
Synthesis of a bridged tricyclic diproline analogue **93** that induces α-helix conformation into linear peptides [90].

The preparation of a bridged tricyclic analogue to induce an α-helix conformation in a linear peptide sequence was accomplished using an anodic oxidation step (Scheme 21). The stabilisation of linear peptides via inducing a stabilised secondary structure is of importance in mimicking protein–protein interactions (PPI) for diseases such as cancer and HIV [89–90]. The methoxylated intermediate **90** was treated with a Lewis acid and vinylmagnesium bromide to afford the *trans*-diastereomer after column chromatography. Coupling of **91** and **92** (prepared using conventional chemistry) resulted in the peptide-turn inducing compound **93**.

**Scheme 22:**
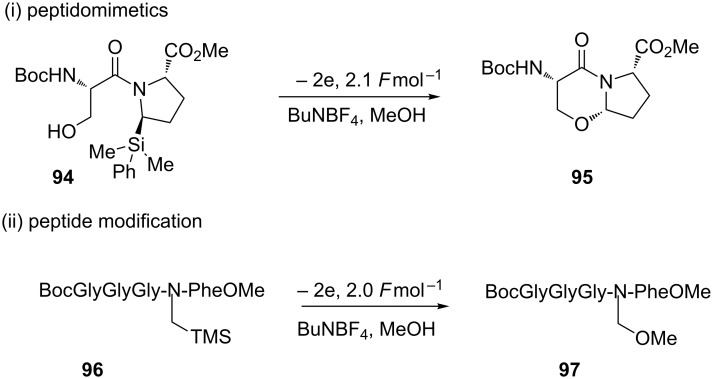
Synthesis of (i) a peptidomimetic and (ii) a functionalised peptide from silyl electroauxiliary precursors [91].

The Moeller research group has carried out extensive research into the synthesis of functionalised peptides and peptidomimetics using the anode oxidation strategy [91–95]. The anodic oxidation of pyrrolidine derivatives and silylated peptides afforded a variety of bicyclic lactam peptidomimetics and functionalised peptides (Scheme 22) [91–92,94]. Constrained amino acid mimics such as **95** are important molecules as they display their functional group in a highly ordered way and can be used to mimic, for example, a proline residue of a natural peptide.

**Scheme 23:**
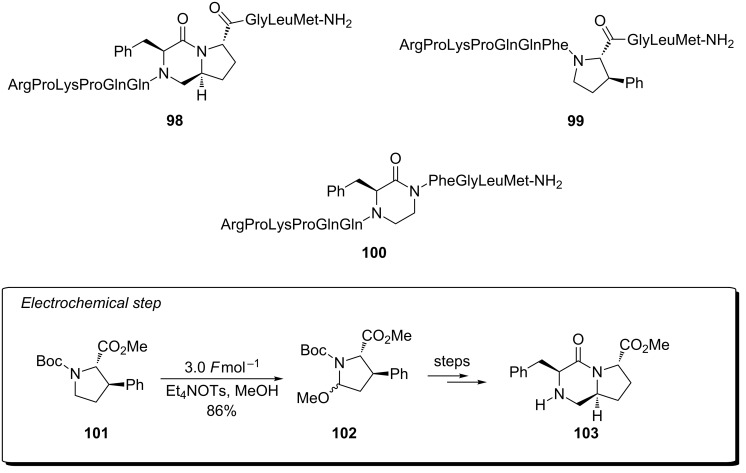
Examples of Phe^7^–Phe^8^ mimics prepared using an electrochemical approach [93].

The Moeller research group has also employed an electrosynthetic approach to the synthesis of a peptidomimetic of substance P [93]. Substance P is an 11 amino acid peptide that contains a phenylalanine^7^–phenylalanine^8^ linkage (Phe^7^–Phe^8^) and a member of the mammalian tachykinin family of peptides implicated in diseases such as arthritis, asthma, inflammatory bowel disease and depression. Electrosynthesis of 3-phenylproline, mono- or bicyclic piperazinone derived cores (conformationally constrained mimics of the Phe^7^–Phe^8^ linkage) afforded the non-natural peptides. These compounds displayed their amino acid residues in similar conformations to the receptor bound conformation of substance P. The three analogues prepared (Scheme 23; **98**–**100**) showed competitive binding of the native ligand with IC_50_’s = 32, 80, 5 µM using a radioiodinated peptide binding to the NK1 receptor in Chinese Hamster Ovary (CHO) cells (native ligand IC_50_ = 0.3 nM).

**Scheme 24:**
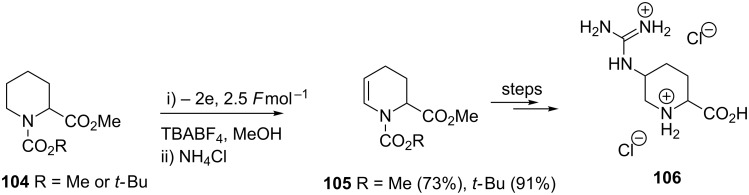
Preparation of arginine mimics employing an electrooxidation step [96].

The preparation of arginine mimetics is an ongoing challenge for chemical biology and epigenetics. Dhimane and co-workers utilised a strategy of anodic methoxylation to complete the first step in the synthesis of **106** (Scheme 24) [96–97].

**Scheme 25:**
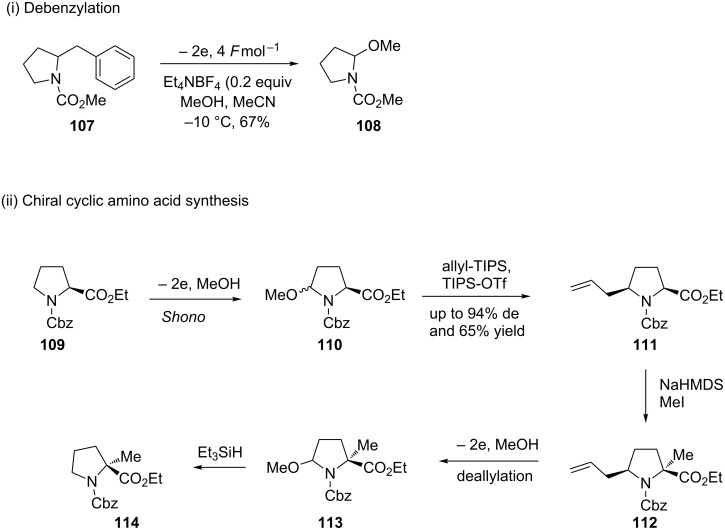
Preparation of chiral cyclic amino acids [20].

The preparation of chiral (up to 99% ee) cyclic amino acids was achieved by Onomura and colleagues (Scheme 25) [20]. In their paper, the authors justify the choice of the anode and cathode material, something not always considered by others working in the field of electrosynthesis, where it was shown that through different combinations of cathode and anodes the product yield was affected and also different amounts of electricity (*F*mol^−1^) was required [20]. The authors also detail a deallylation and a debenzylation deprotection method to **108**. An alternative strategy to chiral amino acids was demonstrated by Kuźnik and colleagues [98] through the electrochemical preparation of 3-triphenylphosphine-2,5-piperazinediones as chiral glycine cation equivalents. Steckhan and co-workers have previously demonstrated the power of electrosynthetic chiral glycine equivalents [99–100].

### Combination of technology and natural product analogue synthesis

**Scheme 26:**
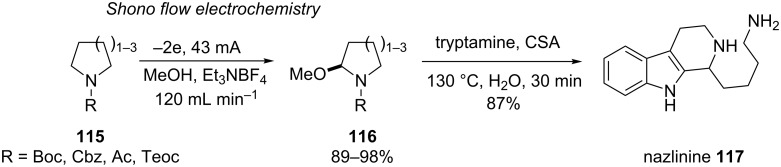
Two-step preparation of Nazlinine **117** using Shono flow electrochemistry [101].

Lastly, Ley and co-workers have recently reported the expedient synthesis of indole alkaloid nazlinine **117** (Scheme 26) using a commercial electrochemical flow cell which allowed the electrolyte loading to be reduced yet still obtained high conversion and product purity [101]. Interestingly, the use of steel or platinum electrode resulted in no conversion to the product and was only possible using a carbon anode. The reason for this was not eluded to and voltammetry was not performed.

The judicial employment of a Shono electrooxidation coupled to a flow cell led to the preparation of a range of α-methoxy cyclic amines in excellent yield. The Ley group then tested this enabling route to the total synthesis of the natural product, nazlinine **117**. Nazlinine was isolated in 1991 from the plant, *Nitraria schoberi*, and exhibits serotonergic bioactivity. There had been only two previous syntheses of nazlinine and neither was sufficiently modular to prepare not only nazlinine but structurally similar analogues. The α-methoxy cyclic amines were treated with tryptamine (or analogues) and a camphorsulfonic acid (CSA)-mediated Pictet–Spengler reaction afforded the desired Nazlinine and structural variants in one pot.

## Conclusion

In this review article we have highlighted the scope of the Shono-type electrooxidation from simple intermediate synthesis to natural product total syntheses and looked at the possibilities of molecularly engineering reaction set-ups to drive the formation of a desired compound electrochemically. We note from the above exciting work that electrochemical parameters and experimental set-ups are in some cases arbitrary with no real consideration or foresight and there remains a great deal further to explore. To quote the namesake of this paper Prof. Tatsuya Shono in his 1984 review “*Since electroorganic chemistry seems rather unfamiliar to those investigating organic synthesis, the purpose of this review is to show that electroorganic chemistry is one of the promising tools for organic synthesis*” [10]. Some excellent progress has been made in the intervening years and still further progress is needed; namely, employing a collaborative approach between synthetic chemists and electrochemists to significantly progress this exciting and burgeoning field.
